# Screen Time at Age 1 Year and Communication and Problem-Solving Developmental Delay at 2 and 4 Years

**DOI:** 10.1001/jamapediatrics.2023.3057

**Published:** 2023-08-21

**Authors:** Ippei Takahashi, Taku Obara, Mami Ishikuro, Keiko Murakami, Fumihiko Ueno, Aoi Noda, Tomomi Onuma, Genki Shinoda, Tomoko Nishimura, Kenji J. Tsuchiya, Shinichi Kuriyama

**Affiliations:** 1 Graduate School of Medicine, Tohoku University, Sendai, Japan; 2 Tohoku Medical Megabank Organization, Tohoku University, Sendai, Japan; 3 Department of Pharmaceutical Sciences, Tohoku University Hospital, Sendai, Japan; 4 United Graduate School of Child Development, Hamamatsu University School of Medicine, Hamamatsu, Japan; 5 Research Center for Child Mental Development, Hamamatsu University School of Medicine, Hamamatsu, Japan; 6 International Research Institute of Disaster Science, Tohoku University, Sendai, Japan

## Abstract

**Importance:**

Whether some domains of child development are specifically associated with screen time and whether the association continues with age remain unknown.

**Objective:**

To examine the association between screen time exposure among children aged 1 year and 5 domains of developmental delay (communication, gross motor, fine motor, problem-solving, and personal and social skills) at age 2 and 4 years.

**Design, Participants, and Setting:**

This cohort study was conducted under the Tohoku Medical Megabank Project Birth and Three-Generation Cohort Study. Pregnant women at 50 obstetric clinics and hospitals in the Miyagi and Iwate prefectures in Japan were recruited into the study between July 2013 and March 2017. The information was collected prospectively, and 7097 mother-child pairs were included in the analysis. Data analysis was performed on March 20, 2023.

**Exposure:**

Four categories of screen time exposure were identified for children aged 1 year (<1, 1 to <2, 2 to <4, or ≥4 h/d).

**Main Outcomes and Measures:**

Developmental delays in the 5 domains for children aged 2 and 4 years were assessed using the Japanese version of the Ages & Stages Questionnaires, Third Edition. Each domain ranged from 0 to 60 points. Developmental delay was defined if the total score for each domain was less than 2 SDs from its mean score.

**Results:**

Of the 7097 children in this study, 3674 were boys (51.8%) and 3423 were girls (48.2%). With regard to screen time exposure per day, 3440 children (48.5%) had less than 1 hour, 2095 (29.5%) had 1 to less than 2 hours, 1272 (17.9%) had 2 to less than 4 hours, and 290 (4.1%) had 4 or more hours. Children’s screen time was associated with a higher risk of developmental delay at age 2 years in the communication (odds ratio [OR], 1.61 [95% CI, 1.23-2.10] for 1 to <2 h/d; 2.04 [1.52-2.74] for 2 to <4 h/d; 4.78 [3.24-7.06] for ≥4 vs <1 h/d), fine motor (1.74 [1.09-2.79] for ≥4 vs <1 h/d), problem-solving (1.40 [1.02-1.92] for 2 to <4 h/d; 2.67 [1.72-4.14] for ≥4 vs <1 h/d), and personal and social skills (2.10 [1.39-3.18] for ≥4 vs <1 h/d) domains. Regarding risk of developmental delay at age 4 years, associations were identified in the communication (OR, 1.64 [95% CI, 1.20-2.25] for 2 to <4 h/d; 2.68 [1.68-4.27] for ≥4 vs <1 h/d) and problem-solving (1.91 [1.17-3.14] for ≥4 vs <1 h/d) domains.

**Conclusions and Relevance:**

In this study, greater screen time for children aged 1 year was associated with developmental delays in communication and problem-solving at ages 2 and 4 years. These findings suggest that domains of developmental delay should be considered separately in future discussions on screen time and child development.

## Key Points

**Question:**

Is there a dose-response association between screen time for children aged 1 year and functional development at ages 2 and 4 years?

**Findings:**

In this cohort study including 7097 mother-child pairs, a dose-response association was observed between greater screen time at age 1 year and developmental delays in communication and problem-solving at ages 2 and 4 years.

**Meaning:**

These findings suggest that domains of developmental delay should be considered separately in future discussions on screen time and child development.

## Teaser

This cohort study examines the association between screen time exposure among children aged 1 year and developmental delay at age 2 and 4 years.

## Introduction

Screen time is the amount of time that individuals spend watching television, playing video games, and using mobile phones, tablets, and other electronic devices. To ensure that children engage in physical activity and obtain adequate sleep for healthy growth and well-being, the World Health Organization^1^ and the American Academy of Pediatrics^2^ have issued guidelines that recommend limiting screen time for children, including a limit of 1 hour per day for children aged 2 to 5 years.^2^ However, a recent meta-analysis reported that only a minority of children meet these guidelines.^3^ In addition, children’s screen time has increased in recent years because of the rapid proliferation of digital devices and the COVID-19 pandemic.^4,5,6^ Therefore, it is essential to consider how screen time affects child development.

Previous studies have reported associations between screen time and child development outcomes. These outcomes include communication,^7,8^ daily living skills,^7^ socialization,^7^ gross and fine motor skills,^8^ problem-solving skills,^8^ personal and social skills,^8^ developmental screening test total scores,^9^ cognitive development,^10,11^ socioemotional development,^9^ language development,^11,12,13^ attention problems,^14^ behavioral problems,^15,16^ and developmental disorders such as autism spectrum disorder.^17,18^

Although several studies have examined the association between screen time and child development outcomes, 2 questions remain. The first is whether screen time is associated with child development domains and, if so, which ones. Because there are several domains of child development, its association with screen time may be domain specific. However, most previous studies examined a single measure as an outcome.^9,10,11,12,13,14,15,16,17,18^ Only 2 studies^7,8^ focused on multiple child development domains: one that considered several domains was cross-sectional,^8^ and the other performed a longitudinal examination.^7^ Therefore, further research focusing on several developmental domains is needed to clarify the association between screen time and individual child development domains.

The second question is whether the association between children’s screen time and developmental delay continues with age. To our knowledge, only 2 studies^9,16^ have examined whether screen time is associated with child development outcomes at several later time points. Both studies used random-intercept cross-lagged panel models: one examined the association between screen time and developmental screening scores at ages 2, 3, and 5 years,^9^ and the other examined the association between screen time and externalizing and internalizing behavior at ages 3, 5, 7, and 9 years.^16^ The findings of both studies did not support an association between children’s screen time at a single point and child development outcomes at 2 or more later points.^9,16^ These studies examined developmental and behavioral screening test scores^9,16^; however, there are several phenotypic domains of child development.

Considering these findings, it is essential to examine whether screen time is continuously associated with child development domains at multiple time points and, if so, which ones. Therefore, this study examined the association between screen time exposure at age 1 year and 5 domains of developmental delay (communication, gross motor, fine motor, problem-solving, and personal and social skills) at ages 2 and 4 years among participants in the Tohoku Medical Megabank Project Birth and Three-Generation (TMM BirThree) Cohort Study, a representative population in Japan and one of the largest cohorts for this research area.

## Methods

### Study Design and Population

Details of the TMM BirThree cohort study are provided elsewhere.^19,20,21,22^ The Tohoku Medical Megabank Organization Institutional Review Board reviewed and approved the study protocol. Pregnant women at 50 obstetric clinics and hospitals in the Miyagi and Iwate prefectures in Japan were recruited into the study between July 2013 and March 2017.^19,20^ Trained genomic medical research coordinators explained the study details to all potential participants and obtained signed consent.^19,20^ The study followed the Strengthening the Reporting of Observational Studies in Epidemiology (STROBE) reporting guideline.

Of the 23 130 mother-child pairs in the TMM BirThree cohort, 16 033 were excluded as follows: 505 withdrew informed consent, 875 participated in the study survey more than once, 8820 were missing information on screen time at age 1 year, and 2512 and 3321 were missing information on development outcomes at ages 2 and 4 years, respectively. Therefore, 7097 mother-child pairs were included in the analysis (Figure).

**Figure.**
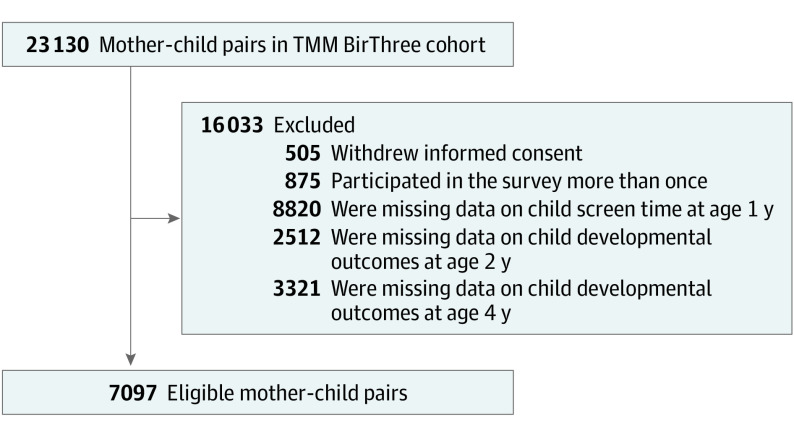
Flowchart of Study Exclusion Criteria TMM BirThree indicates Tohoku Medical Megabank Project Birth and Three-Generation.

### Screen Time

Children’s screen time at age 1 year was assessed using a questionnaire in which participants were asked the following: “On a typical day, how many hours do you allow your children to watch TV, DVDs, video games, internet games (including mobile phones and tablets), etc?” There were 5 response categories: none, less than 1, 1 to less than 2, 2 to less than 4, or 4 or more hours per day. We merged 2 categories (none and <1), resulting in 4 categories of screen time exposure (<1, 1 to <2, 2 to <4, or ≥4 h/d).

### Child Development

To assess developmental delay among children, we used the Ages & Stages Questionnaires, Third Edition (ASQ-3).^23,24^ The ASQ-3 assesses child development from ages 1 to 66 months. In this study, parents responded to questions in the Japanese version of the ASQ-3 regarding their children aged 2 and 4 years.^24^ The ASQ-3 comprised 6 questions divided into the following 5 domains: communication (babbling, vocalizing, and understanding), gross motor (arm, body, and leg movement), fine motor (hand and finger movement), problem-solving (learning and playing with toys), and personal and social skills (solitary social play and playing with toys and other children). The response options included “yes,” “sometimes,” or “not yet” (10, 5, or 0 points, respectively), and each domain was scored with a range of 0 to 60 points.^23,24^ If 1 or 2 of the 6 questions were missed, the remaining total score was multiplied by 1.2 or 1.5, adjusted from 0 to 60, respectively.^23,24^ One question in the gross motor domain for children aged 2 years asked about a possible behavior that they may have had previously but no longer did because they acquired more advanced skills. If parents answered “not yet” or “sometimes” on the easier item and “yes” on the more advanced item, the response on the earlier item was changed to “yes.”^23,24^ A total score of each domain that was less than −2 SDs relative to the mean in reference indicated developmental delay and the need for further assessment.^23,24^ A previous study showed that this cutoff point had moderate sensitivity and specificity to estimate any delay, severe delay, motor delay, and cognitive delay,^25^ and it has also been used widely in the screening of Japanese children.^26,27^

### Covariates

We selected covariates that may affect the association between children’s screen time and developmental delay based on previous studies.^7,8,9,10,11,12,13,14,15,16,17,18^ Children’s sex was garnered from birth records. Information about maternal age at delivery and parity (nulliparous, or primiparous or multiparous) was gathered from medical records. We divided maternal age into 4 categories (<25, 25-29, 30-35, or >35 years). Information on annual household income (<¥4 000 000 [US <$28 400], ¥4 000 000-5 999 999 [US $28 400-$42 599], or ≥¥6 000 000 [US ≥$42 600]) was gathered from the midpregnancy questionnaire. Data on maternal educational attainment (high school graduate or less, junior college or vocational college graduate, university graduate or above, or other), child living with grandparents or other adults (yes or no), and maternal postpartum depression and maternal bonding disorder were gathered using the questionnaire at 1 year post partum. Maternal postpartum depression was assessed using the Japanese version of the Edinburgh Postnatal Depression Scale (EPDS).^28,29,30^ In Japan, an EPDS score of 9 or higher is widely used as the cutoff point for screening of postpartum depression, with previous studies reporting sensitivity of 75% and 82% and specificity of 93% and 95% at 1 month post partum.^29,30^ Maternal bonding disorder was assessed using the Japanese version of the Mother-to-Infant Bonding Scale^31,32,33^ (MIBS-J), and the cutoff point was set at 5. A previous study of Japanese mothers with 1-month-old infants showed that an MIBS-J cutoff point of 4 or 5 correctly classified approximately 90% of pathological maternal bonding disorders.^33^

### Statistical Analysis

Participant characteristics were described according to the 4 categories of child screen time at age 1 year (<1, 1 to <2, 2 to <4, or ≥4 h/d). Characteristics are presented as frequencies with percentages and as medians with IQRs. Associations between the 4 screen time categories at 1 year and the 5 ASQ-3 domains of developmental delay in children at 2 and 4 years were evaluated using multivariable logistic regression analysis to estimate odds ratios (ORs) and 95% CIs (with <1 h/d as the reference). Missing covariates were imputed through multiple imputations by chained equations using the exposure, outcome, and covariates in the main analysis.^34^ Twenty sets of quasi-complete data were analyzed in the multivariable analyses independently and the estimates were integrated.^34^ In addition, as a supplemental analysis, a complete case analysis was performed in which participants with at least 1 missing covariate were excluded. All statistical analyses were performed using R, version 4.0.2 (R Project for Statistical Computing), and 95% CIs not crossing 1.00 were considered statistically significant. Data analysis was performed on March 20, 2023.

## Results

### Study Population Characteristics

Of the 7097 children included this study, 3674 were boys (51.8%) and 3423 were girls (48.2%). Table 1 presents participant characteristics according to the 4 categories of children’s screen time. In terms of screen time exposure per day, 3440 children (48.5%) had less than 1 hour, 2095 (29.5%) had 1 to less than 2 hours, 1272 (17.9%) had 2 to less than 4 hours, and 290 (4.1%) had 4 or more hours. At age 2 years, developmental delays were observed in the communication (361 [5.1%]), gross motor (400 [5.6%]), fine motor (329 [4.6%]), problem-solving (301 [4.2%]), and personal and social skills (387 [5.5%]) domains. At age 4 years, developmental delays were also observed in the communication (283 [4.0%]), gross motor (303 [4.3%]), fine motor (349 [4.9%]), problem-solving (269 [3.8%]), and personal and social skills (328 [4.6%]) domains. Mothers of children with high levels of screen time were characterized as being younger, having never given birth, and having a lower household income, lower maternal education level, and having postpartum depression.

**Table 1.** Participant Characteristics According to Screen Time at Age 1 Year^a^

Characteristic	Total (N = 7097)	Screen time at age 1 y, h/d			
		<1 (n = 3440)	1 to <2 (n = 2095)	2 to <4 (n = 1272)	≥4 (n = 290)
Child sex					
Male	3674 (51.8)	1844 (53.6)	1053 (50.3)	623 (49.0)	154 (53.1)
Female	3423 (48.2)	1596 (46.4)	1042 (49.7)	649 (51.0)	136 (46.9)
Child living with grandparents or other adults at 1 y post partum					
Yes	1516 (21.4)	781 (22.7)	448 (21.4)	235 (18.5)	52 (17.9)
No	5581 (78.6)	2659 (77.3)	1647 (78.6)	1037 (81.5)	238 (82.1)
Maternal age at delivery, y					
<25	329 (4.6)	135 (3.9)	105 (5.0)	65 (5.1)	24 (8.3)
25-29	1635 (23.0)	714 (20.8)	527 (25.2)	328 (25.8)	66 (22.8)
30-35	2740 (38.6)	1355 (39.4)	779 (37.2)	495 (38.9)	111 (38.3)
>35	2393 (33.8)	1236 (35.9)	684 (32.6)	384 (30.2)	89 (30.6)
Parity					
Nulliparous	3363 (47.4)	1331 (38.7)	1084 (51.7)	755 (59.4)	193 (66.6)
Primiparous or multiparous	3715 (52.3)	2099 (61.0)	1006 (48.1)	515 (40.5)	95 (32.7)
Missing	19 (0.3)	10 (0.3)	5 (0.2)	2 (0.2)	2 (0.7)
Annual household income, ¥^b^					
<4 000 000	2294 (32.3)	1039 (30.2)	670 (32.0)	468 (36.8)	117 (40.3)
4 000 000-5 999 999	2274 (32.0)	1043 (30.3)	706 (33.7)	429 (33.7)	96 (33.1)
≥6 000 000	2219 (31.3)	1223 (35.6)	619 (29.5)	316 (24.8)	61 (21.0)
Missing	310 (4.4)	135 (3.9)	100 (4.8)	59 (4.7)	16 (5.6)
Maternal educational attainment					
High school graduate or less	2097 (29.5)	971 (28.2)	616 (29.4)	408 (32.1)	102 (35.2)
Junior college or vocational college graduate	2775 (39.1)	1323 (38.5)	823 (39.3)	510 (40.1)	119 (41.0)
University graduate or above	2155 (30.4)	1110 (32.3)	635 (30.3)	342 (26.9)	68 (23.4)
Other	18 (0.3)	8 (0.2)	5 (0.2)	5 (0.4)	0 (0)
Missing	52 (0.7)	28 (0.8)	16 (0.8)	7 (0.5)	1 (0.4)
Maternal postpartum depression					
Yes	854 (12.0)	367 (10.7)	256 (12.2)	186 (14.6)	45 (15.5)
No	6230 (87.8)	3063 (89.0)	1838 (87.8)	1084 (85.2)	245 (84.5)
Missing	13 (0.2)	10 (0.3)	1 (0)	2 (0.2)	0 (0)
EPDS score, median (IQR)	4.0 (3.0-6.0)	4.0 (2.0-6.0)	4.0 (3.0-7.0)	5.0 (3.0-7.0)	4.0 (3.0-7.0)
Bonding disorder					
Yes	782 (11.0)	311 (9.0)	260 (12.4)	174 (13.7)	37 (12.8)
No	6232 (87.8)	3086 (89.8)	1811 (86.5)	1087 (85.4)	248 (85.5)
Missing	83 (1.2)	43 (1.2)	24 (1.1)	11 (0.9)	5 (1.7)
MIBS-J score, median (IQR)	1.0 (0-3.0)	1.0 (0-2.0)	1.0 (0-3.0)	1.0 (0-3.0)	1.0 (0-3.0)
ASQ-3 domain, median (IQR)					
Age 2 y					
Communication	55 (40-60)	55 (45-60)	55 (40-60)	50 (35-60)	50 (25-60)
Gross motor	60 (50-60)	60 (50-60)	60 (50-60)	60 (50-60)	60 (50-60)
Fine motor	50 (50-55)	50 (50-55)	50 (45-55)	50 (45-55)	50 (45-55)
Problem-solving	50 (45-60)	55 (45-60)	50 (45-60)	50 (40-60)	50 (40-55)
Personal and social skills	50 (45-50)	50 (45-50)	50 (45-50)	50 (45-50)	45 (40-50)
Age 4 y					
Communication	60 (55-60)	60 (55-60)	60 (55-60)	60 (55-60)	60 (50-60)
Gross motor	60 (55-60)	60 (55-60)	60 (55-60)	60 (54-60)	60 (50-60)
Fine motor	55 (50-60)	60 (50-60)	55 (50-60)	55 (50-60)	55 (45-60)
Problem-solving	60 (55-60)	60 (55-60)	60 (55-60)	60 (55-60)	60 (50-60)
Personal and social skills	60 (50-60)	60 (50-60)	60 (50-60)	60 (50-60)	55 (50-60)
Developmental delay					
Age 2 y					
Communication	361 (5.1)	119 (3.5)	112 (5.3)	87 (6.8)	43 (14.8)
Gross motor	400 (5.6)	187 (5.4)	119 (5.7)	69 (5.4)	25 (8.6)
Fine motor	329 (4.6)	145 (4.2)	99 (4.7)	62 (4.9)	23 (7.9)
Problem-solving	301 (4.2)	116 (3.4)	89 (4.2)	67 (5.3)	29 (10.0)
Personal and social skills	387 (5.5)	169 (4.9)	106 (5.1)	80 (6.3)	32 (11.0)
Age 4 y					
Communication	283 (4.0)	115 (3.3)	74 (3.5)	69 (5.4)	25 (8.6)
Gross motor	303 (4.3)	143 (4.2)	93 (4.4)	48 (3.8)	19 (6.6)
Fine motor	349 (4.9)	163 (4.7)	101 (4.8)	64 (5.0)	21 (7.2)
Problem-solving	269 (3.8)	129 (3.8)	59 (2.8)	60 (4.7)	21 (7.2)
Personal and social skills	328 (4.6)	142 (4.1)	102 (4.9)	63 (5.0)	21 (7.2)

Abbreviations: ASQ-3, Ages & Stages Questionnaires, Third Edition; EPDS, Edinburgh Postnatal Depression Scale; MIBS-J, Mother-to-Infant Bonding Scale, Japanese version.

^a^ Unless indicated otherwise, values are presented as No. (%) of participants.

^b^ To convert Japanese yen to US dollars, multiply by 0.0071.

### Multivariable Logistic Regression Analysis for Screen Time and Developmental Delay Among Children

Table 2 presents the association between the 4 screen time categories at age 1 year and each domain of developmental delay at ages 2 and 4 years through multivariable logistic regression (with <1 h/d as the reference). After adjusting for covariates, we observed an association between screen time at age 1 year and a higher risk of developmental delay at age 2 years in the communication (OR, 1.61 [95% CI, 1.23-2.10] for 1 to <2 h/d; 2.04 [1.52-2.74] for 2 to <4 h/d; 4.78 [3.24-7.06] for ≥4 vs <1 h/d), fine motor (1.74 [1.09-2.79] for ≥4 vs <1 h/d), problem-solving (1.40 [1.02-1.92] for 2 to <4 h/d; 2.67 [1.72-4.14] for ≥4 vs <1 h/d), and personal and social skills (2.10 [1.39-3.18] for ≥4 vs <1 h/d) domains. We also observed an association between screen time at age 1 year and developmental delay at age 4 years in the communication (OR, 1.64 [95% CI, 1.20-2.25] for 2 to <4 h/d; 2.68 [1.68-4.27] for ≥4 vs <1 h/d) and problem-solving (1.91 [1.17-3.14] for ≥4 vs <1 h/d) domains.

**Table 2.** Association of Participants’ Screen Time With Developmental Delay

ASQ-3 domain and screen time at age 1 y, h	OR (95% CI)			
	Age 2 y		Age 4 y	
	Crude	Adjusted^a^	Crude	Adjusted^a^
Communication				
<1	1 [Reference]	1 [Reference]	1 [Reference]	1 [Reference]
1 to <2	1.58 (1.21-2.05)	1.61 (1.23-2.10)	1.06 (0.78-1.42)	1.08 (0.80-1.46)
2 to <4	2.05 (1.54-2.72)	2.04 (1.52-2.74)	1.66 (1.22-2.24)	1.64 (1.20-2.25)
≥4	4.86 (3.32-7.00)	4.78 (3.24-7.06)	2.73 (1.70-4.21)	2.68 (1.68-4.27)
Gross motor				
<1	1 [Reference]	1 [Reference]	1 [Reference]	1 [Reference]
1 to <2	1.05 (0.83-1.33)	0.98 (0.77-1.25)	1.07 (0.82-1.40)	1.03 (0.78-1.35)
2 to <4	1.00 (0.75-1.32)	0.90 (0.68-1.21)	0.90 (0.64-1.25)	0.82 (0.58-1.16)
≥4	1.64 (1.04-2.49)	1.46 (0.93-2.28)	1.62 (0.96-2.59)	1.44 (0.87-2.40)
Fine motor				
<1	1 [Reference]	1 [Reference]	1 [Reference]	1 [Reference]
1 to <2	1.13 (0.87-1.46)	1.07 (0.82-1.39)	1.02 (0.79-1.31)	0.98 (0.76-1.28)
2 to <4	1.16 (0.85-1.57)	1.06 (0.77-1.45)	1.07 (0.79-1.43)	0.99 (0.73-1.35)
≥4	1.96 (1.21-3.03)	1.74 (1.09-2.79)	1.57 (0.95-2.46)	1.35 (0.83-2.21)
Problem-solving				
<1	1 [Reference]	1 [Reference]	1 [Reference]	1 [Reference]
1 to <2	1.27 (0.96-1.68)	1.17 (0.88-1.55)	0.74 (0.54-1.01)	0.74 (0.53-1.01)
2 to <4	1.59 (1.17-2.16)	1.40 (1.02-1.92)	1.27 (0.92-1.73)	1.23 (0.89-1.70)
≥4	3.18 (2.05-4.81)	2.67 (1.72-4.14)	2.00 (1.21-3.16)	1.91 (1.17-3.14)
Personal and social skills				
<1	1 [Reference]	1 [Reference]	1 [Reference]	1 [Reference]
1 to <2	1.03 (0.80-1.32)	0.98 (0.76-1.26)	1.19 (0.91-1.54)	1.17 (0.90-1.53)
2 to <4	1.30 (0.98-1.70)	1.19 (0.90-1.58)	1.21 (0.89-1.63)	1.13 (0.83-1.55)
≥4	2.40 (1.59-3.53)	2.10 (1.39-3.18)	1.81 (1.10-2.85)	1.60 (0.98-2.61)

Abbreviations: ASQ-3, Ages & Stages Questionnaires, Third Edition; OR, odds ratio.

^a^ Adjusted for maternal age at delivery, parity, maternal educational attainment, household income, child sex, living with grandparents or other adults at 1 year post partum, bonding disorder at 1 year post partum, and postpartum depression at 1 year post partum.

We conducted a supplemental analysis that excluded 19 children whose parents self-reported that their child had been diagnosed with autism spectrum disorder and cerebral palsy by age 4 years as a factor in the association between screen time and developmental delay. We observed that the estimates did not show any meaningful departure from the main results.

The eTable in Supplement 1 presents the results of the complete case analysis. No significant difference in interpretation due to the use of multiple imputations was observed.

## Discussion

The findings of this study support previous research showing an association between screen time among young children and subsequent developmental outcomes.^7,8,9,10,11,12,13,14,15,16,17,18^ These results also suggest that there was a dose-response association between longer screen time at age 1 year and developmental delays in communication and problem-solving at ages 2 and 4 years. In particular, more than 4 hours of screen time per day was associated with developmental delays in communication and problem-solving across ages 2 and 4 years.

The association observed between screen time and developmental delay among young children was domain specific. For example, the associations between screen time of children aged 1 year and the communication and problem-solving domains were consistent across ages, although no association was observed in the gross motor domain at ages 2 and 4 years. Sugiyama et al^7^ examined the association between screen time at age 2 years and 3 domains (communication skills, daily living skills, and social skills) at age 4 years. They found that screen time was associated with poorer communication and daily living skills and was not associated with social skills.^7^ In terms of domain-specific associations, their results are consistent with ours. Here, associations were consistently observed in the communication and problem-solving domains for children aged 2 and 4 years and not in the personal and social skills domain at age 4 years. In addition, a meta-analysis^12^ reported an association between screen time and language development, and a cross-sectional study^8^ examining the association between screen time and the 5 domains of the ASQ-3 found associations in the domains of communication, problem-solving, and personal and social skills. The results of these previous studies and our study suggested an association between screen time and communication and problem-solving domains in young children, while results for personal and social skills were inconsistent across studies. Based on 2 longitudinal time points of outcomes for each developmental domain, this study emphasized that screen time was not associated with all developmental domains.

We observed that screen time for children aged 1 year was associated with the fine motor and personal and social skills domains at age 2 years; however, this association was not confirmed at age 4 years. There are 2 possible hypotheses for this finding. One hypothesis is that the developmental delay of fine motor and personal and social skills for children aged 2 years caught up with them at age 4 years. Further follow-up studies would be needed to verify whether this phenomenon is specific to the fine motor and personal and social skills domains or whether the association is not confirmed with age even in the communication and problem-solving domains. Another hypothesis is that reverse causation occurs, in which a developmental delay of fine motor or personal and social skills lengthens screen time. Previous studies have examined the association between screen time and the personal and social skills domain; although a cross-sectional study reported an association,^8^ a prospective study found no association.^7^ Application of the reverse causality described earlier would explain why the association was confirmed in the cross-sectional study and not in the prospective study. However, our prospective study confirmed an association between screen time at age 1 year and developmental delay of personal and social skills at age 2 years. Although this phenomenon is unknown, applying the reverse causality hypothesis may explain the confirmed association at age 2 years in our study by assuming that developmental delay in the personal and social skills domain at age 1 year leads to longer screen time at age 1 year, and developmental delay in the personal-social domain at age 1 year is reflected in the developmental delay in that domain at age 2 years.

Although screen time has been associated with developmental delay, it may have an educational aspect depending on the programs watched on electronic devices. In fact, a meta-analysis showed that greater screen use was associated with decreased language skills, whereas screen time spent on educational programs was associated with increased language skills.^12^ In addition, the American Academy of Pediatrics recommends that high-quality (eg, educational) programs should be selected when introducing digital media to children aged 18 to 24 months.^2^ Because it is difficult to limit screen time in general in today’s world of electronic devices, it may be beneficial to identify and limit the screen time aspects that are associated with developmental delays while taking advantage of the educational aspects.

### Strengths and Limitations

This study has 2 strengths. First, developmental delay was measured using the ASQ-3, which has been validated worldwide and used in a variety of studies.^9,35,36,37^ Although the ASQ-3 is not a diagnostic tool, it is an appropriate screening tool for examining developmental delays according to several developmental domains. Second, the analysis was conducted with one of the largest prospective cohorts of any study examining the association between screen time and child development outcomes.

A limitation is that the information we collected did not allow us to separate educational screen time from other types of screen time. Doing so may have helped us in examining the association between screen time and child development while considering both positive and negative aspects of screen time.

## Conclusions

In this cohort study, greater screen time at age 1 year was associated in a dose-response manner with developmental delays in communication and problem-solving at ages 2 and 4 years. These findings suggest that domains of developmental delay should be considered separately in future discussions on screen time and child development.

## Notes

Supplement 1. **eTable.** Results of the Complete Case Analysis (n = 6656)

Supplement 2.
Data Sharing Statement
