# Supplementary material for: Screen Time at Age 1 Year and Communication and Problem-Solving Developmental Delay at 2 and 4 Years
Source: JAMA Pediatr. 2023 Aug 21;177(10):1039–46. doi: 10.1001/jamapediatrics.2023.3057 (PMC10442786; doi:10.1001/jamapediatrics.2023.3057)
Supplement: Supplement 1. — eTable. Results of the Complete Case Analysis (n = 6656) [file jamapediatr-e233057-s001.pdf]

Supplementary Online Content

Takahashi I, Obara T, Ishikuro M, et al. Screen time at age 1 year and communication and problem-solving developmental delay at 2 and 4 years. *JAMA Pediatr*. Published online August 21, 2023. doi:10.1001/jamapediatrics.2023.3057

eTable. Results of the Complete Case Analysis (n = 6656)

This supplementary material has been provided by the authors to give readers additional information about their work.

© 2023 American Medical Association. All rights reserved.

eTable. Results of the Complete Case Analysis (n = 6656)

ASQ-3	Screen time at 1 year old	2 years old	4 years old
		OR* (95％CI)	OR* (95％CI)
Communication	<1	Ref	Ref
	1-<2	1.70 (1.28-2.25)	1.11 (0.80-1.51)
	2-<4	2.13 (1.56-2.89)	1.72 (1.24-2.38)
	≥4	5.00 (3.29-7.47)	2.66 (1.59-4.28)
Gross motor	<1	Ref	Ref
	1-<2	1.05 (0.82-1.33)	1.02 (0.76-1.35)
	2-<4	0.90 (0.66-1.21)	0.81 (0.56-1.15)
	≥4	1.45 (0.89-2.25)	1.52 (0.88-2.51)
Fine motor	<1	Ref	Ref
	1-<2	1.06 (0.80-1.40)	0.98 (0.74-1.29)
	2-<4	1.05 (0.76-1.45)	0.99 (0.72-1.36)
	≥4	1.52 (0.89-2.47)	1.28 (0.74-2.12)
Problem-solving	<1	Ref	Ref
	1-<2	1.21 (0.90-1.62)	0.78 (0.56-1.08)
	2-<4	1.39 (0.99-1.92)	1.18 (0.83-1.66)
	≥4	2.47 (1.52-3.87)	1.95 (1.13-3.21)
Personal and social skills	<1	Ref	Ref
	1-<2	1.03 (0.79-1.34)	1.20 (0.91-1.58)
	2-<4	1.23 (0.91-1.64)	1.18 (0.85-1.61)
	≥4	2.06 (1.32-3.14)	1.52 (0.87-2.50)

*Adjusted for maternal age at delivery, parity, maternal educational attainment, household income, children's sex, living with grandparents or other adults at 1 year postpartum, bonding disorder at 1 year postpartum and postpartum depression at 1 year postpartum.
Missing covariates were imputed through multiple imputations by chained equation using the exposure, outcome, and covariates.

© 2023 American Medical Association. All rights reserved.
