# Supplementary material for: Screen Time at Age 1 Year and Communication and Problem-Solving Developmental Delay at 2 and 4 Years
Source: JAMA Pediatr. 2023 Aug 21;177(10):1039–46. doi: 10.1001/jamapediatrics.2023.3057 (PMC10442786; doi:10.1001/jamapediatrics.2023.3057)
Supplement: Supplement 2. — Data Sharing Statement [file jamapediatr-e233057-s002.pdf]

Data Sharing Statement

Takahashi. Screen Time at Age 1 Year and Communication and Problem-Solving Developmental Delay at 2 and 4 Years. *JAMA Pediatr*. Published August 21, 2023. doi:10.1001/jamapediatrics.2023.3057

Data
Data available: Yes
Data types: Deidentified participant data
How to access data: The data that support the findings of this study are available from the TMM biobank; however, restrictions apply to the availability of these data, which were used under license for the current study and hence are not publicly available. Data are available from the authors upon reasonable request and with the permission of the TMM biobank. All inquiries about access to the data should be sent to the TMM biobank (dist@megabank.tohoku.ac.jp).
When available: With publication

Supporting Documents
Document types: None

Additional Information
Who can access the data: Researchers whose proposed use of the data has been approved
Types of analyses: For any purpose
Mechanisms of data availability: After approval of a proposal
